# Prevalence of *Helicobacter pylori* infection among resettled refugees presenting to a family medicine clinic in the United States

**DOI:** 10.1111/hel.12894

**Published:** 2022-04-28

**Authors:** Nadia Saif, Nicole Jensen, Elizabeth Farrar, Sarah Blackstone, Fern R. Hauck

**Affiliations:** ^1^ University of Maryland School of Medicine University of Maryland at Baltimore Baltimore Maryland USA; ^2^ Department of Family Medicine University of Virginia Health System Charlottesville Virginia USA

**Keywords:** antibiotic therapy, epidemiology, *helicobacter pylori* infection, prevalence, refugees, treatment failure

## Abstract

**Background:**

Although endemic to much of the global population, few studies have examined *Helicobacter pylori* (*H. pylori)* in US refugee populations. This study investigates the prevalence of *H. pylori* infection and barriers to treatment in the International Family Medicine Clinic (IFMC), a primary care refugee clinic, in central Virginia.

**Materials and Methods:**

We conducted a chart review of 188 refugee patients of the IFMC who were referred for an *H. pylori* test between January 1, 2019, and December 31, 2020. Recorded measures included patient demographics, *H. pylori* test result, treatment of initial infection, completion of test of cure (TOC), TOC results, salvage therapy, and barriers to treatment. Binary logistic regression was performed to examine the association between demographic factors and *H. pylori* test results.

**Results:**

Of the 171 patients who completed an *H. pylori* test, 94 tested positive (54.9%). Of the 93 patients that were subsequently treated, 76 were treated with clarithromycin triple therapy (82%). Forty‐eight patients (52%) completed a TOC after completing treatment, and 21 (43%) of these patients remained positive, indicating persistent infection. Eighteen patients (90%) who remained positive for *H. pylori* were subsequently treated with quadruple therapy. Patients under 18 (OR = 0.25, *p* < 0.01) and patients with a history of previous *H. pylori* (OR = 0.44, *p* < 0.05) were less likely to have positive results on initial *H. pylori* testing. Common barriers to treatment included pregnancy, religious observance (e.g., fasting), and health system complications (e.g., prior authorization for medications, cost of treatment).

**Conclusions:**

The prevalence of *H. pylori* among refugees at the IFMC was higher than the overall prevalence estimate for the United States, which is consistent with previous studies. This work represents an updated picture of *H. pylori* prevalence among refugees in the United States and contributes to the identification of treatment barriers.

## Significance statement

This article describes a chart review of recently resettled adult and children refugees treated at the main family medicine clinic at a large University‐based medical center from 2019 to 2020. Results include the prevalence of *H. pylori* infection among those tested, treatment resistance based on patients that required additional rounds of treatment for eradication, and identified barriers to treatment completion. This study adds to the scant literature on epidemiology of *H. pylori* infection among refugee populations in the United States, especially considering the importance of refugee and immigrant populations to the burden of *H. pylori* infection in North America.

## 1 INTRODUCTION

*Helicobacter pylori* (*H. pylori*) is a gram‐negative, rod‐shaped bacterium that infects the epithelial lining of the stomach and underlies one of the most common bacterial infections in humans.^1^, ^2^ Over half of the global population is infected, with the highest estimated prevalence in Africa (79.1%), Latin America and the Caribbean (63.4%), and Asia (54.7%), compared with lower prevalence in Northern America (37.1%) and Oceania (24.4%).^3^ Infection is usually acquired in childhood. Risk factors include lower socioeconomic status or social disadvantage, male sex, and having multiple siblings (due to intra‐familial spread).^2^ It is thought that exposure to contaminated water is a possible source of transmission in developing countries.

In North America, prevalence is higher among certain racial and ethnic groups, including African Americans, Hispanic Americans, Native Americans, Canadian First Nations populations, and Alaska natives.^2^ Immigrants to North America, especially those from Asia, Africa, and Central and South America, also have higher prevalence rates than those from North America. Additionally, those living near the United States (U.S.) border with Mexico are a higher‐risk group.^2^ Few studies have reported the prevalence of *H. pylori* in refugee populations worldwide, although prevalence in refugee source countries is high.^3^, ^4^, ^5^ This is in contrast to the estimated prevalence of 35% in the U.S. general population based on a 2017 systematic review by Hooi et al.^3^ The top origin countries for refugees resettled in the United States in 2020 were as follows: the Democratic Republic of Congo, Myanmar (Burma), Ukraine, Afghanistan, Iraq, and Syria; over the long term (2001–2019), most refugees have originated from Myanmar, Iraq, and Somalia.^6^

While *H. pylori* prevalence has been overall decreasing in Western nations, immigrants and refugees are thought to have a prevalence similar to their counterparts in their native countries.

Although usually asymptomatic, *H. pylori* is considered a major cause of peptic ulcer disease (PUD) and gastritis. Treatment is important because those infected have an increased risk of developing gastric cancer and mucosal associated‐lymphoid‐type (MALT) lymphoma;^1^ thus, all symptomatic adults found to have active infection should be offered treatment.^2^ Furthermore, understanding common barriers to treatment and gaps in *H. pylori* treatment can guide future efforts to ensure refugee patients have access to appropriate care. In developing countries, most infections are acquired in childhood by the age of 5 years. Some children may spontaneously clear the infection but become reinfected. However, reinfection is less likely in children who acquired the initial infection in developed countries.^7^ Among children, treatment is only recommended after a careful discussion of the risks and benefits with parents, as treatment is unlikely to improve abdominal symptoms other than those due to PUD, and there is a lower risk of progression to chronic complications compared with adults.^7^, ^8^ Aside from symptomatic PUD, testing for *H. pylori* infection has roles in the assessment of other conditions including refractory iron‐deficiency anemia and immune thrombocytopenic purpura (ITP).^8^ Testing can also be considered prior to starting chronic non‐steroid anti‐inflammatory drug (NSAID) therapy.^2^

Screening for asymptomatic *H. pylori infection* is not currently routine in Western countries, despite some evidence that it may be cost‐effective in certain populations. A study assessing the cost‐effectiveness of screening high‐risk refugee and immigrant populations found that use of stool antigen testing through a general screening strategy, followed by treatment of positive cases, then retesting to confirm cure, is relatively cost‐effective considering the number of gastric cancer and peptic ulcer cases averted.^9^ This held true even at an *H. pylori* population prevalence as low as 25%.^9^

Noninvasive diagnostic tests to confirm active infection include the urea breath test (UBT; FDA‐approved for above age 3 years, with 85%–95% sensitivity and 85%–100% specificity) and fecal antigen test (94% sensitivity and 97% specificity regardless of age).^7^ Both tests can also be used for confirming eradication after treatment and should be completed at least 4 weeks following antibiotics and 2 weeks after discontinuation of a proton pump inhibitor (PPI), due to potential for false negatives in presence of PPIs, or recent use of antibiotics or bismuth preparations. Serologic testing is not recommended for diagnostic nor eradication testing due to persistence of IgG antibodies long after infection has cleared, and lower reliability in children. However, as serologic testing is widely available, inexpensive, and less cumbersome for patients than UBT and fecal antigen testing, it may have a role in screening among high‐risk populations.^2^, ^10^

Selection of a treatment regimen is guided by prior antibiotic exposure and information on prevalence of clarithromycin resistance. Treatment failure is common due to increasing resistance of clarithromycin, metronidazole, and fluoroquinolones.^2^ First‐line triple therapy using a PPI, clarithromycin, and amoxicillin can be used. Due to increasing rates of clarithromycin resistance, however, the 2017 American College of Gastroenterology Clinical Guideline recommends bismuth quadruple therapy which includes a PPI, bismuth, metronidazole, and tetracycline as the preferred first‐line regimen for most patients.^2^ Among children, susceptibility testing is recommended prior to treatment. For strains that are clarithromycin‐resistant or susceptibility is unknown, the recommended triple therapy regimen is PPI, amoxicillin, and metronidazole (or bismuth if metronidazole resistance is also unknown).^7^

Because of the limited information on prevalence of *H. pylori*, treatment completion, and barriers to treatment in refugee populations, this study sought to address the following objectives: (1) estimate the prevalence and success of the treatment of *H. pylori* in refugee adult and pediatric patients attending a refugee primary care clinic, and (2) identify barriers to treatment. Given the potential long‐term complications of *H. pylori*, this research can offer valuable insight to guide initiatives linking refugee patients to *H. pylori* treatment.

## 2 MATERIALS AND METHODS

### 2.1 Study setting and participants

The University of Virginia (UVA) Family Medicine clinic uniquely houses the International Family Medicine Clinic (IFMC). The IFMC serves as the primary care home for refugees and Special Immigrant Visa (SIV) holders (hereafter referred to as refugees) resettled in Charlottesville, VA, and the surrounding area. Since 2002, the clinic has served more than 4000 patients. The patient population hails from various countries including Afghanistan, the Democratic Republic of Congo, Nepal, Somalia, Myanmar, Iraq, and Syria. All refugee patients attending the clinic undergo a set of screening laboratory tests upon arrival. *H. pylori* testing is not included in this initial universal screening, but is ordered when clinical indications are identified at patient visits.

The IFMC manages a database in which all patients seen at the clinic have an “IFMC” flag in their chart. Using clinical encounter reports, we identified patients who had been referred for an *H. pylori* test between 2019 and 2020 using the electronic medical record (EMR). Patients were included in the study if they had an IFMC flag in the chart *and* if they had been referred for an *H. pylori* test during the study time period. The study was approved by the University of Virginia Health Sciences Institutional Review Board (IRB) and determined to be exempt from full IRB review, and therefore, informed consent was not required.

### 2.2 Data collection

We obtained the following information from the EMR: age, sex, country of origin, country of exit, whether the patient had spent time in a refugee camp, primary language spoken, and previous history of *H. pylori* infection. Country of origin (the patient’s country of birth) and country of exit (the country the patient left immediately prior to resettlement in the United States) were re‐coded into the following regions: Central Asia, Eastern Europe, Middle East, South America, and Southeast Asia. Three investigators conducted a chart review to determine the type of *H. pylori* test ordered, initial test result, initial treatment (triple therapy, quadruple therapy or other), completion of test of cure (TOC), TOC results, follow‐up treatment, and follow‐up TOC (if applicable). *H. pylori* testing included urea breath testing (UBT), stool antigen testing, and serology. Triple therapy included omeprazole 20 mg BID, clarithromycin 500 mg BID, and amoxicillin 1 g BID (or metronidazole 500 mg BID for penicillin‐allergic) for 14 days. Quadruple therapy included omeprazole 20 mg BID, bismuth subcitrate 420 mg QID, metronidazole 250 mg QID, and tetracycline 500 mg QID for 14 days.

### 2.3 Data analysis

Frequencies were obtained for patient characteristics including age group, sex, region of origin, region of exit, experience in refugee camp (yes/no), language (English/non‐English), history of previous infection, *H. pylori* test result, treatment of initial infection, and completed TOC.

To simultaneously examine the influence of demographic factors on *H. pylori* test result (positive or negative), binary logistic regression was conducted, controlling for sex, age group, history of previous infection, language, and whether patient spent time in a refugee camp. Patients who had not completed their *H. pylori* test were removed due to low numbers. A second logistic regression was used to examine predictors of the need for a second course of antibiotics, controlling for sex, age group, history of previous infection, language, and whether patient spent time in a refugee camp. A subset of patients who tested positive on the initial *H. pylori* test *and* who completed the initial TOC were included. A positive TOC result was used to indicate whether a second round of antibiotics was needed. Analyses were conducted in R 4.0.5 using the *modelr* package.

For all patients that tested positive for *H. pylori*, clinic visit notes were reviewed to extract information on any mentioned barriers that may have impacted completion of treatment. These barriers were reviewed and summarized.

## 3 RESULTS

In total, 188 IFMC patients were referred for an *H. pylori* test during the study period and 171 patients completed testing. Table 1 presents characteristics of all patients and patients who initially tested positive for *H. pylori*. Out of 171 patients who completed testing, 54.9% (*n* = 94) tested positive for *H. pylori,* and nearly all of these patients were prescribed treatment (*n* = 93). Of patients who tested positive initially, 36% (*n* = 33) had a history of prior *H. pylori* infection. Among adults with completed tests, 60.0% (*n* = 84) tested positive compared with 32.3% (*n* = 10) among children. The majority of patients were prescribed triple antibiotic therapy (*n* = 76; 82%), which was the recommended first‐line treatment regimen at the IFMC at the time the study was conducted. A much smaller number were prescribed quadruple therapy (*n* = 15; 16%). Two patients were prescribed regimens other than triple therapy or quadruple therapy. This included one patient who was prescribed lansoprazole, tinidazole, clarithromycin, and omeprazole, which was a continuation of a regimen prescribed before arriving to the United States; another patient was prescribed pantoprazole only for symptom management due to current pregnancy.

**TABLE 1:** Characteristics of Patients with *H. pylori* Tests Ordered, 2019–2020, *n* (column %)

Characteristic	Negative, *N* = 77	Positive, *N* = 94	Chi‐Squared Statistic*
Age group			8.87*
Under 18	21/(27%)	10/(11%)	
18–39	32/(42%)	55/(59%)	
40–64	23/(30%)	28/(30%)	
65 and over	1/(1.3%)	1/(1.1%)	
Sex			0.01
Female	47/(61%)	56/(60%)	
Male	30/(39%)	38/(40%)	
Region of origin			4.21
Africa	5/(6.6%)	10/(11%)	
Central Asia	58/(76%)	64/(71%)	
Eastern Europe	0/(0%)	1/(1.1%)	
Middle East	4/(5.3%)	8/(9.0%)	
North America	1/(1.3%)	0/(0%)	
Southeast Asia	8/(11%)	7/(7.9%)	
Region of exit			5.43
Africa	5/(6.7%)	9/(9.9%)	
Central Asia	48/(64%)	52/(56%)	
Eastern Europe	0/(0%)	1/(1.1%)	
Middle East	12/ (176)	19/(21%)	
South America	0/(0%)	3/(3.3%)	
Southeast Asia	10/(13%)	8/(8.8%)	
Spent time in refugee camp			1.36
Yes	10/(13%)	18/(20%)	
No	56/(73%)	61/(65%)	
Unknown	11/(14%)	14/(15%)	
Language			0.67
English	17/(22%)	15/(16%)	
Non‐English	60/(78%)	79/(84%)	
Previous Infection			
Yes	35 (55%)	33 (35%)	4.45*
No/Unknown	31 (45%)	61 (65%)	
Treatment of initial infection (N = 93)			‐‐‐
Triple therapy^a^	‐‐‐	76 (82%)	
Quadruple therapy^b^	‐‐‐	15 (16%)	
Other^c^	‐‐‐	2 (2.2%)	
Had test of cure (TOC)^d^			
Yes	‐‐‐	48 (52%)	
No	‐‐‐	45 (48%)	

Omeprazole 20 mg BID, clarithromycin 500 mg BID, and amoxicillin 1 g BID (or metronidazole 500 mg BID for penicillin‐allergic) for 14 days.

Omeprazole 20 mg BID, bismuth subcitrate 420 mg QID, metronidazole 250 mg QID, and tetracycline 500 mg QID for 14 days.

One patient was prescribed a regimen of lansoprazole, tinidazole, clarithromycin, and omeprazole which was a continuation of a regimen prescribed before arriving to the U.S.; another patient was prescribed pantoprazole only for symptom management due to current pregnancy.

Of those that completed treatment.

*p < 0.05.

Among patients who completed testing, the overwhelming majority were from Afghanistan (country of origin for *n* = 122, 71%, and country of exit for *n* = 94, 56%), followed by Turkey (country of exit for *n* = 16, 9.5%), Democratic Republic of Congo (DRC; country of origin for *n* = 12, 7%), Nepal (country of exit for *n* = 11, 6.5%), Bhutan (country of origin for *n* = 9, 5.3%), and smaller numbers for Burundi, Tanzania, Iran, Iraq, Lebanon, Syria, India, Pakistan, Myanmar, Rwanda, Sudan, Colombia, Russia, and Thailand. Of note, some of these countries overlapped in patients' country of origin vs. exit; for example, most patients with country of origin Bhutan exited via Nepal, and this was similarly noted for DRC (country of origin) and Tanzania (country of exit).

The region of origin with the highest prevalence of *H. pylori* in our sample (defined as positive test results among all patients from that region tested) was Africa (*n* = 10 patients tested positive; 66.7%), followed by the Middle East (*n* = 8, 66.7%), Central Asia (*n* = 64, 57.7%), and Southeast Asia (*n* = 7, 46.7%). A similar pattern was evident for prevalence by region of exit: Africa was highest (*n* = 9, 64.3%), followed by the Middle East (*n* = 19, 63.3%), Central Asia (*n* = 52, 57.1%), and then Southeast Asia (*n* = 8, 47.1%).

The results and outcomes of the TOC are presented in Table 2. Of the patients who tested positive, 48 patients (52%) completed a TOC after completing treatment. Of patients who completed the initial TOC, 21 (43%) tested positive for *H. pylori* a second time and the majority of these patients were prescribed quadruple antibiotic therapy (*n* = 18; 90%).

**TABLE 2:** Outcome of Test of Cure (TOC) Among Patients who Completed *H. pylori* Treatment, 2019–2020, *n* (%)

Characteristic	Patients who Completed Treatment and TOC after Initial Infection *N* = 48^a^
TOC result	
Negative	27 (57%)
Positive	21 (43%)
2nd treatment regimen (of positive TOC patients)	
Triple therapy^b^	1 (5.0%)
Quadruple therapy^c^	18 (90%)
Other^d^	1 (5.0%)
2nd TOC	
Yes	13 (65%)
No	8 (35%)
2nd TOC result	
Negative	11 (85%)
Positive	2 (15%)

Only one patient who tested positive on the second TOC received a third round of treatment.

Omeprazole 20 mg BID, clarithromycin 500 mg BID, and amoxicillin 1 g BID for 14 days.

Omeprazole 20 mg BID, bismuth subcitrate 420 mg QID, metronidazole 250 mg QID, and tetracycline 500 mg QID for 14 days.

Amoxicillin 500 mg TID and Omeprazole 40 mg TID for 14 days, prescribed by Gastroenterology.

Of patients referred for initial *H. pylori* testing, patients under age 18 were significantly less likely to have a positive test compared to patients age 18–39 (adjusted OR = 0.25, 95% CI 0.09, 0.67, *p* < .01; Table 3). Additionally, patients with a history of previous infection were less likely to have a positive result on the initial test (adjusted OR = 0.44, 95% CI, 0.20, 0.94, *p* < .05; Table 3). Among patients who initially tested positive *and* completed a TOC, patients who had a history of previous infection had greater odds of needing a second course of antibiotics (adjusted OR = 6.09, 95% CI, 1.47, 30.20, *p* < .05; Table 4).

**TABLE 3:** Multivariable Logistic Regression of Demographic Factors Tested for Association with *H. pylori* Infection among Patients with Test Completed Between 2019–2020

Characteristic	Adjusted^a^ Odds Ratio (95% Confidence Interval)
Sex	
Female	Reference
Male	1.24 (0.55, 2.82)
Age group	
18–39	Reference
**Under 18**	0.25 (0.09, 0.67)**
40–64	1.08 (0.43, 2.81)
65 and over	0.55 (0.02, 14.90)
Language	
English	Reference
Non‐English	1.24 (0.42, 3.59)
Previous infection	
No	Reference
**Yes**	0.44 (0.20, 0.94)*
Spent time in refugee camp	
No	Reference
Yes	0.96 (0.36, 2.67)

Adjusted for sex, age group, history of previous infection, language and whether patient spent time in a refugee camp.

*p < 0.05, **p < 0.01.

**TABLE 4:** Multivariable Logistic Regression of Demographic Factors Tested for Association with Requiring Salvage Therapy among Patients Treated and Completed Test of Cure (TOC), 2019–2020

Characteristic	Adjusted^a^ Odds Ratio (95% Confidence Interval)
Sex	
Female	Reference
Male	1.22 (0.26, 6.03)
Age group	
18–39	Reference
40–64	1.86 (0.34, 10.30)
65 and over	N/A
Under 18	0.21 (0.01, 2.53)
Language	
English	Reference
Non‐English	0.33 (0.03, 2.82)
Previous infection	
No	Reference
**Yes**	6.09 (1.47, 30.20)*
Spent time in refugee camp	
No	Reference
Yes	1.16 (0.20, 6.80)

Adjusted for sex, age group, history of previous infection, language and whether patient spent time in a refugee camp.

*p < 0.05.

Several trends emerged among the 45 patients with an initial positive test who did not complete a TOC (48%). In 2019, per the chart review, a TOC was not discussed with the patient by their provider for many patients with an initial positive test. Additionally, some providers ordered a TOC which was not completed for various reasons, including an inability to contact the patient to ensure completion of the test. For patients with an initial positive test in 2020, several lacked a TOC because the study period for review ended on December 31, 2020; 5 of these patients had a TOC completed in 2021. Other reasons for not completing a TOC included intolerance to treatment and subsequent discontinuation of therapy, and delayed TOC in favor of continuing PPI use. Many patients reported satisfactory symptom resolution, so providers deferred a test to confirm complete clearance of the infection. The impact of the COVID‐19 pandemic on missed appointments or delayed follow‐up after treatment potentially contributed to lack of TOC for some patients as well.

Our chart review highlighted several common barriers to treatment or eradication of infection. Fifteen patients were found to have a family member with a concurrent *H. pylori* infection in which eradication was only achieved once the entire family was treated, the barrier being likely reinfection within the household. Pregnancy was the second most common barrier we identified, as many patients delayed treatment until after delivery. Several patients delayed treatment for various other reasons, such as to observe religious fasts for the Islamic holy month of Ramadan. Other patients delayed returning to the clinic for a TOC due to concerns about COVID‐19. Health system complications such as prior authorization requirements for medications, a laboratory specimen error (and therefore testing was not completed), medication costs, and inability to contact a patient for follow‐up were additional barriers noted. Furthermore, no interpreter services were available for one patient, requiring her to rely upon her spouse for interpretation. Other barriers included side effects of treatment, missed follow‐up appointments, and concerns about patients not taking the medications as directed. Notably, some patients reported that they were infected or reinfected while visiting their home country during recent travel.

## 4 DISCUSSION

Our study found that the prevalence of *H. pylori* among refugee patients of all ages who completed a noninvasive diagnostic *H. pylori* test between 2019 and 2020 presenting to the IFMC in Charlottesville, Virginia, was 54.9%. Though inclusion criteria specified that a refugee be tested for one of the following tests (UBT, stool antigen, or serology), none of the patients included in the study were tested for *H. pylori* with serology, and therefore, we can assume that this result reflects active *H. pylori* infection. It should be noted that at the IFMC, *H. pylori* testing is not included in routine screening and generally only ordered if a patient is symptomatic or as confirmation of eradication after treatment.

The overall prevalence of *H. pylori* was higher among adults than children (60.0% vs. 32.3%, respectively), and odds of infection were lower among patients less than 18 years of age. The prevalence of *H. pylori* infection in refugee children at the IFMC was found to be consistent with the global prevalence of childhood *H. pylori* infection (32.6%).^11^ A history of prior infection with *H. pylori* was also found to be protective against initial infection, but was a risk factor for requiring salvage therapy after a positive test of cure. We are uncertain what to make of this relationship given our current data, but a possibility is that those with prior infection were more likely to have been treated for *H. pylori* in the past and thus eradicated their infection, but conversely, having a history of infection also raises likelihood of having a resistant infection if not treated appropriately initially. There were no significant associations between infection and region of origin or exit, having spent time in a refugee camp, or language spoken.

Our prevalence estimate is similar to the global estimated prevalence for *H. pylori*, and higher than known estimated prevalence for the U.S. population overall. This is consistent with the understanding that immigrants from developing nations, including refugee populations who are more often subject to poor living conditions, have a higher prevalence of *H. pylori* infection than their counterparts in Western host nations.^2^, ^12^ Our prevalence estimates for the refugee regions of origin of Africa (66.7%) and Central Asia (57.7%), however, are lower than previously published estimates in a recent global systematic review (79.1% for Africa and 79.5% for Central Asia)^3^. This is likely explained by our small sample size, and relatively large contribution by a select few countries to our regions of origin (e.g., Afghanistan was the only country in our Central Asia region, while the previously published estimate was based on Kazakhstan only). Our prevalence of 46.7% for the Southeast Asia region of origin, however, is similar to that of the previously published estimate^3^. Our study adds to the scant literature on *H. pylori* infection prevalence among refugee populations who have migrated to North America. In terms of other regions, a few studies have investigated *H. pylori* prevalence among refugees in Australia. A study of 922 refugee adults and children attending the Migrant Health Service in South Australia identified a prevalence of 21.5% by fecal antigen testing,^13^ though it should be noted that in this study *H. pylori* screening was completed on arrival and testing was not based on symptoms. A much higher prevalence of 82% for fecal antigen testing was found among 192 African refugee children resettled in Australia in 2006.^14^

There was a significant prevalence of treatment failure among our sample, as nearly half of those who completed treatment had a positive TOC. There exist several possible contributing factors for this high rate of treatment failure. Most patients were initially prescribed clarithromycin‐based triple therapy followed by quadruple therapy as salvage therapy after a positive TOC; there may have been a high prevalence of clarithromycin‐resistant strains of *H. pylori* among our patient population. Clarithromycin‐based triple therapy was the recommended first‐line regimen at the IFMC at the time of this study, which has subsequently changed due to the results of this study, other treatment failures, and the mounting literature on resistance rates. We identified a combination of individual, community, and system‐based factors as barriers to successful treatment for those with a positive *H. pylori* test. Addressing these barriers is important to help with treatment completion rates among refugee patients, who already struggle with health access barriers related to health literacy and linguistic and cultural factors, which make adherence to a complicated regimen with multiple antibiotics even more challenging. Addressing this will be key to preventing development of treatment‐resistant infections.

Strengths of our study include use of an EMR database that contains relatively complete information on all refugees seen in the IFMC. The IFMC is where most recently resettled refugees and Special Immigrant Visa holders in Charlottesville, Virginia, receive their primary medical care, so our data capture most refugees who present with symptoms concerning for *H. pylori* infection in this community. Thus, these data provide a reliable estimate of *H. pylori* prevalence among refugees within this particular community in the United States.

Limitations of our study include its cross‐sectional nature which precludes ability to assess temporality and cause/effect between the risk factors investigated and *H. pylori* infection. While the IFMC database is relatively complete as mentioned above, it is possible that some refugee patients that did not have the “IFMC” flag in their chart were inadvertently missed and not included. This is rare and sometimes occurs if patients request that the flag be removed or if they moved to regular Family Medicine care outside of the IFMC (e.g., if they have been living in the United States for a long period of time), or it is inadvertently removed for other reasons such as during EMR updates. Further, we did not include data for diagnosis of *H. pylori* based solely on endoscopy. Selection bias is also present, as this study was limited to one specific clinic population within a specific timeframe. Additionally, initial *H. pylori* testing during the study period was ordered only for symptomatic patients, as universal testing of refugee patients upon arrival is not the current standard at the IFMC. Therefore, our prevalence estimates represent positive testing among symptomatic patients only and not among the refugee clinic population overall, and are therefore biased (likely higher than the true prevalence among refugees). The chart review was conducted during a period which included the start of the COVID‐19 pandemic. The early period of the pandemic was associated with reduction in clinic volume, appointment cancelations, switch from in‐person to telemedicine visits, and reduction in new refugee patients being resettled, among other disruptions to normal clinic and community operations. Therefore, COVID‐19 was likely associated with delays in identification of *H. pylori* infection, prescription of treatment, and obtaining tests of cure.

This study highlights the need for future research among refugee populations in the United States regarding *H. pylori* infection and the specific factors associated with treatment failure and resistance. In particular, our findings illustrate two important points: There was a high rate of persistent infection after using clarithromycin‐based triple therapy, and test of cure to confirm eradication was performed on a low proportion of patients. Efforts to reduce use of clarithromycin‐based regimens in areas of high resistance based on updated reports of resistance among isolates in the United States^15^ and more universal eradication testing after treatment completion would likely translate to higher rates of cure in this population. Treatment of *H. pylori* is challenging at baseline, given complicated regimens with multiple antibiotics that carry risk of side effects, coupled with rising resistance to commonly prescribed regimens. Refugee patients often experience the additional burdens of language barriers, health literacy concerns, and less familiarity with the U.S. health system among other factors, which make successful treatment exceedingly challenging. The high prevalence of infection we identified also raises the question about whether universal screening for *H. pylori* infection among refugees upon arrival could be a supported strategy, and further research would help clarify this.

## 5 CONCLUSION

Our study found that the prevalence of *H. pylori* among refugees at a primary care refugee clinic was higher than the overall prevalence estimate for the United States, which is consistent with previous studies. This work represents an updated picture of *H. pylori* prevalence among refugees in the United States and contributes to profile development of patients with positive *H. pylori* tests and completion of TOC, as well as the identification of treatment barriers. A focus on transitioning from clarithromycin‐based triple therapy to regimens with lower resistance rates based on regional data, improving the rate of patients tested for eradication of *H. pylori* after treatment, and addressing the specific social and health literacy barriers that affect refugee populations are all important in increasing cure rates.

## 7 CONFLICT OF INTEREST

The authors have no conflicts of interest to disclose. No external funding sources were used for this study.

## 8 AUTHOR CONTRIBUTIONS

The specific contributions of each author are as follows: All authors made substantial contributions to conception and design of the study and were involved in drafting the manuscript or revising it critically for important intellectual content; gave final approval of the version to be published and participated sufficiently in the work to take public responsibility for appropriate portions of the content; agreed to be accountable for all aspects of the work in ensuring that questions related to the accuracy or integrity of any part of the work are appropriately investigated and resolved. N. Saif, N. Jensen, E. Farrar, and S. Blackstone contributed to acquisition of the data and interpretation of the data. S. Blackstone conducted the data analysis.

9

## Data Availability

The data that support the findings of this study are available from the corresponding author upon reasonable request.
